# A P2RX2 exon-2 complex indel disrupts splicing and reduces P2X2 output: implications for DFNA41 and variable expressivity

**DOI:** 10.3389/fgene.2026.1934538

**Published:** 2026-10-20

**Authors:** Yilun Tao, Wenxia Song, Lihong Wang, Tongli Han, Dong Han, Luna Hao, Chen Zhao, Jiangyuan Ping, Xiaoze Li

**Affiliations:** 1 Medical Genetic Center, Changzhi Maternal and Child Health Care Hospital, Changzhi, Shanxi, China; 2 Precision Medicine Research Division, Changzhi Maternal and Child Health Care Hospital, Changzhi, Shanxi, China; 3 Department of Pediatrics, Changzhi Maternal and Child Health Care Hospital, Changzhi, Shanxi, China; 4 Department of Neurology, National Center for Children’s Health, Beijing Children’s Hospital, Capital Medical University, Beijing, China

**Keywords:** aberrant splicing, DFNA41, minigene, modifier effects, *P2RX2*

## Abstract

**Background:**

*P2RX2* encodes the ATP-gated P2X2 receptor associated with autosomal dominant non-syndromic hearing loss 41 (DFNA41). DFNA41 is characterized by progressive sensorineural hearing loss and an increased susceptibility to acoustic trauma. However, the specific phenotypic consequences of truncating or splice-altering variants within this gene remain poorly understood.

**Methods:**

We evaluated a Han Chinese family using a targeted hearing-loss gene panel with segregation analysis. Splicing consequences of a complex indel in *P2RX2* exon 2 (NM_174873: c.278_288delinsACGTGAAGCCCCC) were assessed using a pcMINI-C minigene assay, followed by RT-qPCR and Western blotting after expression of the reconstructed aberrant isoforms in heterologous cells.

**Results:**

The proband had early-onset severe-to-profound sensorineural hearing loss and carried a heterozygous *P2RX2* complex indel inherited from the father, together with a maternally inherited pathogenic *GJB2* frameshift. Minigene assays revealed cryptic splice usage that generated two frameshifted isoforms with premature termination codons. Both mutant isoforms showed reduced steady-state mRNA and markedly reduced P2X2 protein output, consistent with decreased overall P2X2 expression.

**Conclusion:**

We functionally establish that this exon-2 complex indel disrupts splicing and markedly reduces P2X2 output. These findings support a mechanism of loss-of-function and haploinsufficiency. The pronounced intrafamilial variability—manifesting as profound early-onset hearing loss in the proband compared to a milder phenotype in the heterozygous father—underscores the variable expressivity and incomplete penetrance characteristic of *P2RX2*-linked DFNA41. Although oligogenic modulation is a plausible conceptual framework, it requires cautious interpretation and independent future validation.

## 1 Introduction

Hearing loss is among the most common sensory deficits in humans, and genetic factors are estimated to account for approximately 50%–60% of cases(Aldè et al., 2023; De Rosa et al., 2024). Autosomal dominant nonsyndromic hearing loss (ADNSHL) accounts for roughly one-fifth of hereditary hearing loss and is characterized by pronounced locus and allelic heterogeneity (Aldè et al., 2023). DFNA41 (Deafness, autosomal dominant 41, #608224) has been attributed to variants in *P2RX2* (*600844), which encodes the ATP-gated P2X2 receptor, a trimeric nonselective cation channel of the P2X family (North, 2002; Yan et al., 2013).

In the cochlea, P2X2 is broadly expressed in sensory hair cells, supporting cells, and spiral ganglion neurons (Housley et al., 1999; Kim et al., 2020). Physiologically, this receptor functions as a protective homeostatic regulator. Intense noise exposure triggers ATP release into the endolymph, activating P2X2-mediated cation conductance. This influx creates an electrochemical shunt that transiently reduces the endocochlear potential, thereby dampening the driving force for hair-cell transduction and preventing excitotoxicity during acoustic stress (Yan et al., 2013). Consistent with this model, animal data suggest that P2X2 is not essential for establishing baseline hearing under quiet conditions, yet appears important for long-term cochlear resilience to acoustic stress (George et al., 2019).

To date, DFNA41 pedigrees supported by convincing segregation and functional validation have most often involved missense variants in *P2RX2* that preserve full-length P2X2 subunits but impair gating/assembly (Yan et al., 2013; Faletra et al., 2014; Moteki et al., 2015; Westermann et al., 2025). In contrast, predicted loss-of-function (LOF) alleles (nonsense/frameshift/splice-site) have been reported in sequencing datasets and newborn genomic screening reports, yet these observations frequently lack structured pedigrees, segregation analyses, or longitudinal audiologic follow-up, leaving their causal contribution to DFNA41 insufficiently defined (Antunes et al., 2024; Wang et al., 2024; Hao et al., 2025). Here, we report a Han Chinese family carrying a complex *P2RX2* indel and integrate family-based genetics with transcript/protein studies to define the molecular consequence of a truncating/splice-disrupting allele and to assess whether reduced P2X2 output may contribute to DFNA41 risk, particularly in the presence of additional genetic or environmental modifiers.

## 2 Materials and methods

### 2.1 Subjects and ethical compliance

A two-generation Han Chinese family segregating suspected autosomal dominant sensorineural hearing loss (SNHL) was recruited *via* a special education school for the deaf. The study cohort comprised four individuals: the affected proband and three phenotypically unaffected first-degree relatives (father, mother, and younger sister). Written informed consent was obtained from all participants or their legal guardians. The study protocol was approved by the Institutional Ethics Committee of Changzhi Maternal and Child Health Care Hospital (Approval No. CZSFYLL2023-007).

Comprehensive clinical evaluations were performed on the proband following a standardized protocol. Otoscopic examinations were conducted to exclude external or middle ear pathologies. Audiological assessments, carried out in a sound-treated room, included distortion product otoacoustic emissions (DPOAE) and click-evoked auditory brainstem responses (ABR).

### 2.2 Genetic analysis

A targeted hereditary hearing loss panel covering 406 genes was used (MyGenostics Inc., Beijing, China). Target-enriched libraries were sequenced on an Illumina HiSeq X Ten platform with 150-bp paired-end reads. Reads were mapped to the human reference genome (hg19/GRCh37) using the Burrows–Wheeler Aligner (BWA); PCR duplicates were removed with Picard. Variant calling was performed with the Genome Analysis Toolkit (GATK), followed by annotation with ANNOVAR. Population allele frequencies and prior reports were assessed using the 1000 Genomes Project, the Exome Aggregation Consortium (ExAC), gnomAD, and the Human Gene Mutation Database (HGMD). Variants were prioritized if they were nonsynonymous or located in splice-proximal regions, including canonical splice-site changes and intronic variants within ±10 bp of exon–intron boundaries; synonymous variants and other exonic variants were excluded unless predicted to affect splicing. Variants were classified under the American College of Medical Genetics and Genomics/Association for Molecular Pathology (ACMG/AMP) framework (Richards et al., 2015).

To assess the *in silico* effect of the P2RX2 complex indel on pre-mRNA splicing, we employed three independent algorithms: SpliceAI (https://spliceailookup.broadinstitute.org/), the Rare Disease Data Center Splicing Calculator (RDDCSC; https://rddc.tsinghua-gd.org/), and the BDGP Splice Site Predictor (https://github.com/bdgp/bdgp-splice-promoter-tools). To determine whether any predicted cryptic splice sites represent recognized mis-splicing events, we cross-referenced the SpliceVault database (Dawes et al., 2023).

### 2.3 Minigene splicing assay

To functionally validate the effect of the *P2RX2* variant on pre-mRNA splicing, we engineered minigene constructs using the pcMINI-C reporter system. The genomic insert spanned *P2RX2* Exon two and its flanking regions, including partial Intron 1, Intron 2, and the downstream Exon three context. Target fragments were amplified from patient and control gDNA *via* nested PCR and cloned into the *Kpn*I/*Xho*I sites of the pcMINI-C vector to yield wild-type (pcMINI-C-*P2RX2*-wt) and mutant (pcMINI-C-*P2RX2*-mut) plasmids.

HEK293T and HeLa cells were transiently transfected with the indicated constructs or an empty vector control. Total RNA was harvested 48 h post-transfection and reverse-transcribed. To analyze splicing patterns, cDNA was amplified using vector-specific primers (pcMINI-C-F/R). Amplicons were resolved by agarose gel electrophoresis. Because the variant-induced transcripts (bands b and c) migrated closely, the mixed RT-PCR products were purified and subjected to TA cloning. Multiple independent positive clones were subsequently selected and analyzed *via* Sanger sequencing to definitively resolve the individual aberrant splice junctions and confirm the distinct transcript isoforms.

### 2.4 *P2RX2* mRNA expression analysis

To model the translational consequences of the splicing defects identified in the minigene assay, we constructed expression vectors using the pCMV-3×Flag-Neo(EGFP) backbone. Of note, to evaluate these downstream protein products independently without the confounding variables of alternative splicing efficiency, these expression vectors were separately generated using intronless cDNA sequences rather than the splicing-competent minigene constructs.

The full-length wild-type (wt) *P2RX2* CDS was cloned into the *Hind*III/*Eco*RI sites of the vector. Using this WT construct as a template, we performed overlap-extension PCR to generate two mutant isoforms that precisely recapitulate the target splicing alterations. These were designated as *mut1* (corresponding to the 19-bp exon two deletion; cDNA change: c.278_309delinsACGTGAAGCCCCCGA, predicted p.Trp93Tyrfs*173) and *mut2* (corresponding to the 34-bp exon two deletion; cDNA change: c.276_309del, predicted p.Trp93Glyfs*26). All constructs were subsequently verified *via* Sanger sequencing to confirm the intended mutagenesis and rule out off-target alterations.

HEK293T cells were cultured in DMEM (Gibco) supplemented with 10% fetal bovine serum (FBS) and 1% penicillin-streptomycin in a humidified atmosphere containing 5% CO_2_ at 37 °C. Transient transfections were performed in 6-well plates when cells reached approximately 80% confluence. Transfection complexes were prepared using Lipofectamine 3000 (Invitrogen) and 2 μg of plasmid DNA per well in Opti-MEM reduced-serum medium, strictly following the manufacturer’s protocol. All experiments were conducted in biological triplicates, and cells were harvested 48 h post-transfection for downstream RNA and protein extraction.

Total RNA was extracted from transfected cells using TRIzol reagent (Invitrogen, USA), strictly following the manufacturer’s instructions. First-strand cDNA was synthesized using the Hifair™ 1st Strand cDNA Synthesis SuperMix (Yeasen Biotechnology, Shanghai, China), which integrates a genomic DNA (gDNA) removal step. Quantitative real-time PCR (qPCR) assays were conducted on a Bio-Rad CFX Connect system. Reactions were prepared in a 10 μL final volume containing the 2× Universal SYBR Green Fast qPCR Mix (Toyobo, Osaka, Japan) and specific primers (listed in Supplementary Table S1). The thermal cycling profile consisted of an initial denaturation at 95 °C for 3 min, followed by 45 cycles of 95 °C for 15 s and 60 °C for 30 s. A melt-curve analysis (55 °C–95 °C) was performed immediately after cycling to verify amplicon specificity. Relative P2RX2 mRNA expression levels were quantified using the 2^−ΔΔCT^ method. All assays were performed in independent biological replicates.

### 2.5 P2X2 protein expression analysis

Transfected HEK293T cells were harvested 48 h post-transfection and lysed in ice-cold RIPA buffer supplemented with a protease inhibitor cocktail. Following centrifugation, protein concentrations were determined *via* BCA assay. Of note, to evaluate the distinct translational products of the out-of-frame deletion isoforms independently, Western blotting was performed using separately generated, intronless cDNA expression constructs (*mut1* and *mut2*) rather than the splicing-competent minigene, and the plasmids were separately transfected into cells. Equal amounts of total protein were denatured, resolved by 10% SDS-PAGE, and electroblotted onto PVDF membranes. Membranes were blocked with 5% non-fat milk and probed overnight at 4 °C with primary antibodies against FLAG and GAPDH. Immunoreactive bands were visualized using enhanced chemiluminescence (ECL) on a Monad QuickChemi 5100 system. Densitometric analysis was performed to quantify relative P2RX2 protein expression, targeting the predicted molecular weights of the Flag-tagged isoforms: ∼47 kDa (wt), ∼32 kDa (*mut1*), and ∼15 kDa (*mut2*). All intensity values were normalized to the corresponding GAPDH levels.

### 2.6 Transcriptomic profiling and network interaction analysis

To delineate the cellular localization of *P2RX2* and *GJB2* within the inner ear, we interrogated single-cell transcriptomic profiles using the gEAR Portal (https://umgear.org/). Specifically, we queried the murine cochlear scRNA-seq dataset (Kelley et al., postnatal cochlear epithelium) to map gene expression across annotated cell clusters. Data visualization, including dot plots and heatmaps, relied on the portal’s native normalization and clustering algorithms. Subsequently, we performed Gene Ontology (GO) enrichment analysis was performed on gene sets associated with *GJB2* (UniProt P29033) and *P2RX2* (UniProt Q9UBL9) (https://www.ebi.ac.uk/QuickGO/annotations). Enriched biological processes were visualized as bubble plots. Finally, to elucidate potential synergistic mechanisms, we reconstructed protein–protein and functional interaction networks using GeneMANIA (https://genemania.org/).

## 3 Results

### 3.1 Clinical and audiological findings

The proband passed newborn hearing screening at 3 days old. Subsequent caregiver concern for reduced responsiveness did not prompt confirmatory audiologic testing, and detailed noise-exposure history was unavailable. At 19 months of age, both ABR and DPOAE testing were abnormal. ABR showed no reproducible responses at stimulus levels up to 97 dB nHL in either ear. DPOAE recordings demonstrated low response levels with S/N ratios below 6 dB at most test frequencies in both ears (Figure 1).

**FIGURE 1:**
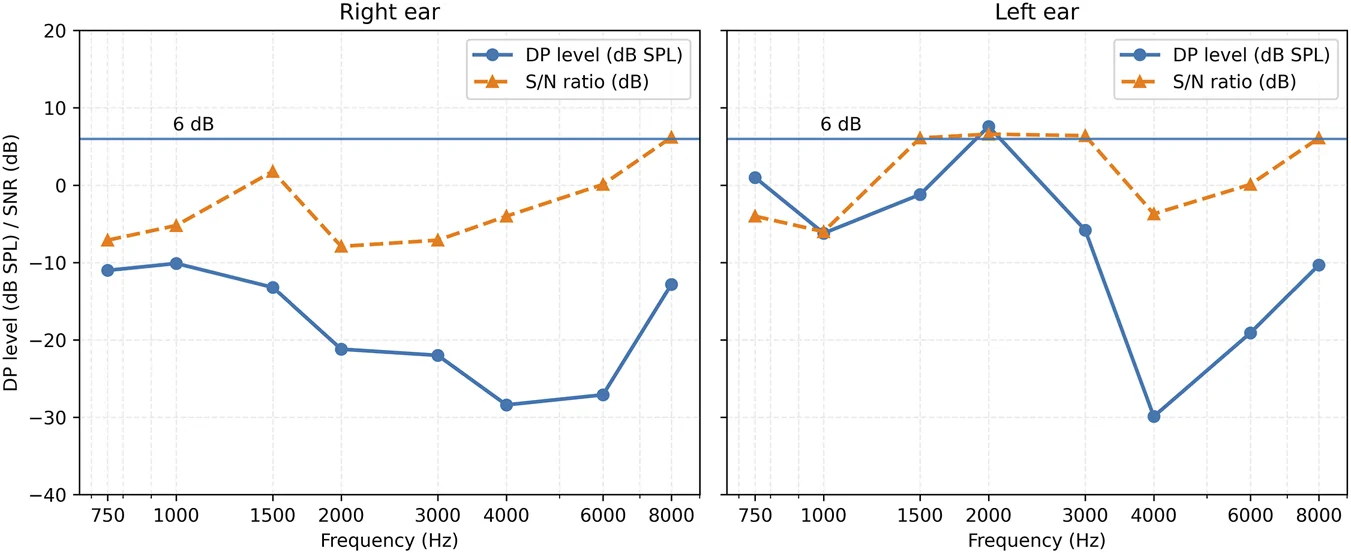
Distortion-product otoacoustic emissions (DPOAEs) assessment at 19 months of age. The profile displays distortion-product (DP) levels (dB SPL; blue circles, solid line) and corresponding signal-to-noise (S/N) ratios (dB; orange triangles, dashed line) across the 0.75–8 kHz frequency range. The horizontal reference line indicates the clinical pass criterion (S/N ≥ 6 dB).

Follow-up assessment at 6 years of age confirmed persistent profound impairment. Click-evoked ABR thresholds exceeded 109 dB nHL bilaterally. Unaided pure-tone audiometry demonstrated severe-to-profound SNHL across all frequencies. Intervention with high-power hearing aids (Phonak Versata SP) provided functional gain, yielding aided thresholds of approximately 50–70 dB HL (Figure 2).

**FIGURE 2:**
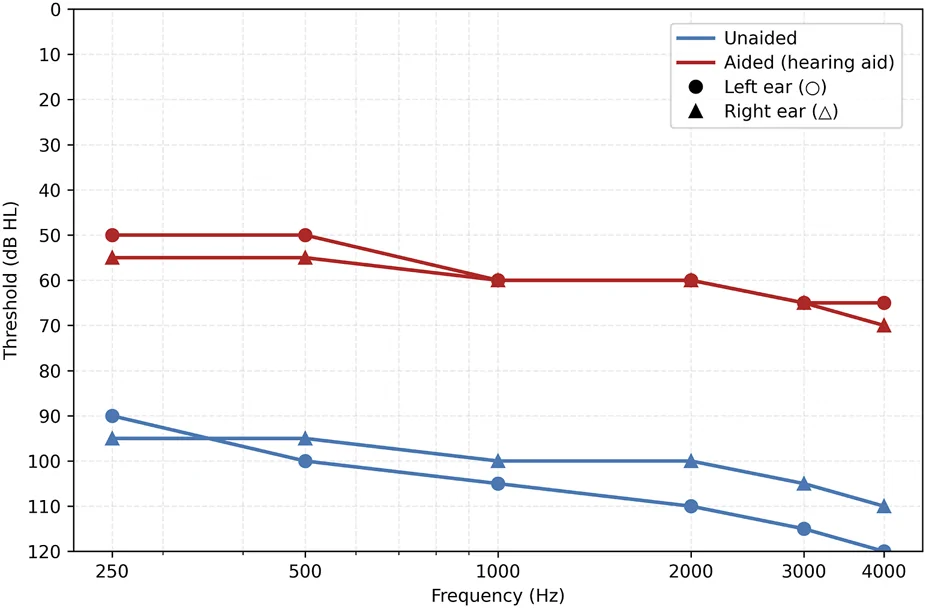
Unaided versus aided pure-tone audiometry at 6 years of age. The audiogram compares air-conduction thresholds in the unaided condition (blue line) against those obtained with high-power hearing aids (Phonak Versata SP; red line). Unaided testing reveals profound bilateral sensorineural hearing loss (90–120 dB HL), while aided responses demonstrate significant functional gain, improving to the 50–70 dB HL range. Markers distinguish left (circles) and right (triangles) ear.

### 3.2 Genetic analysis findings

Targeted genomic sequencing *via* a 406-gene deafness panel revealed a complex genotype involving variants in both *P2RX2* and *GJB2* in the proband. First, a heterozygous complex indel was identified in *P2RX2* exon 2 (NM_174873: c.278_288delinsACGTGAAGCCCCC). High-depth sequencing validation (mean coverage >1000×; Figure 3A) confirmed its authenticity, and the variant was absent from large-scale population databases (e.g., gnomAD). In parallel, the proband harbored the recurrent pathogenic *GJB2* (NM_004004) frameshift c.235delC (p.Leu79Cysfs*3) (Figure 3B). Segregation analysis revealed distinct inheritance trajectories: the *P2RX2* allele was paternally inherited, whereas the *GJB2* variant was present in the mother and younger sister.

**FIGURE 3:**
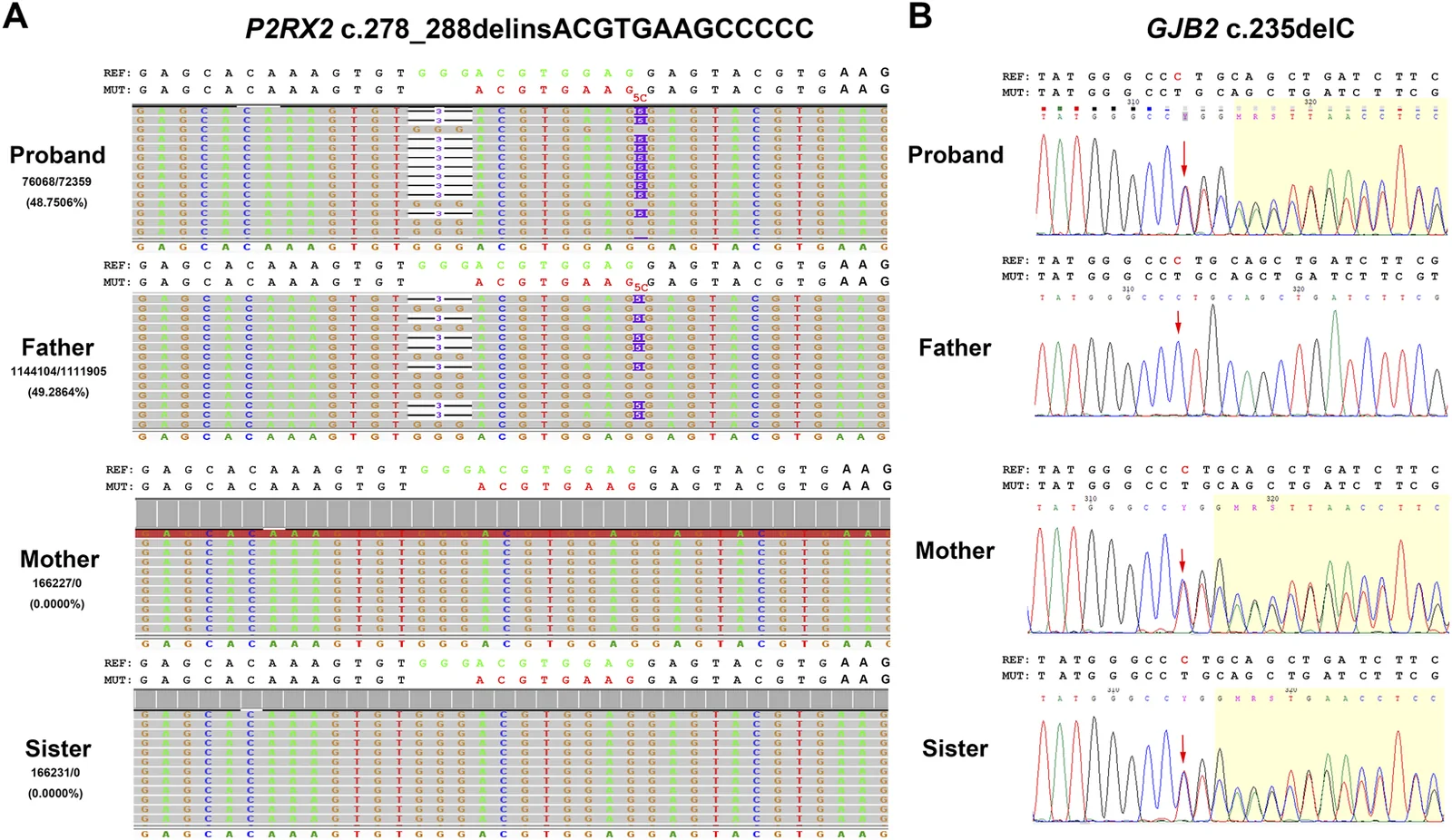
Identification and segregation of variants in *P2RX2* and *GJB2*. **(A)** Deep sequencing reads demonstrate a heterozygous complex indel in *P2RX2* exon 2 (c.278_288delinsACGTGAAGCCCCC). The alignment view specifically highlights a stretch of five consecutive cytosine bases **(C)** within the inserted sequence, marked by the numeral “5”. Segregation analysis confirms paternal transmission: the variant is shared by the proband and the father (allelic fraction ∼50%) but is absent in the mother and younger sister. **(B)** Sanger sequencing chromatograms confirm the *GJB2* variant c.235delC (p.Leu79Cysfs*3). The proband, mother, and younger sister are identified as heterozygous carriers, while the father is wild-type.

According to the standard ACMG/AMP guidelines for variant interpretation, the *P2RX2* complex indel (c.278_288delinsACGTGAAGCCCCC) is classified as Pathogenic (PVS1+PS3+PM2_Supporting). In addition, the GJB2 c.235delC (p.Leu79Cysfs3) variant is a well-established recurrent mutation and is classified as Pathogenic (PVS1+PM3_Verystrong + PM2_Supporting + PP5+BS1).

### 3.3 Minigene splicing analysis

To evaluate whether the *P2RX2* c.278_288delinsACGTGAAGCCCCC variant alters pre-mRNA splicing, we used pcMINI-C minigene reporters spanning *P2RX2* exons 2-3 with flanking intronic sequences (Figure 4A). In both HEK293T and HeLa cells, the wt construct yielded a single RT-PCR product (band a) (Figure 4B). The structural configurations of the canonical and aberrant transcripts are schematically illustrated in Figure 4C. In contrast to wt, the mutant reporter produced two distinct RT-PCR products (bands b and c) in both cell lines, indicating variant-induced aberrant splicing (Figure 4B). Sanger sequencing of these gel-extracted products (Figure 4D) verified the expected exon-exon junctions and mapped both abnormal isoforms to 3′truncations of exon 2: band b harbored a 19-bp deletion [ExonA(192 bp)-Exon2(117 bp)-Exon3(72 bp)], whereas band c harbored a 34-bp deletion [ExonA(192 bp)-Exon2(102 bp)-Exon3(72 bp)]. At the cDNA level, these corresponding aberrant transcripts were cloned into expression vectors designated as *mut1* (band b: c.278_309delinsACGTGAAGCCCCCGA; predicted p.Trp93Tyrfs*173) and *mut2* (band c: c.276_309del; predicted p.Trp93Glyfs*26). Both isoforms result in out-of-frame deletions that are predicted to introduce premature termination codons, and the same aberrant splicing pattern was observed across both cell lines.

**FIGURE 4:**
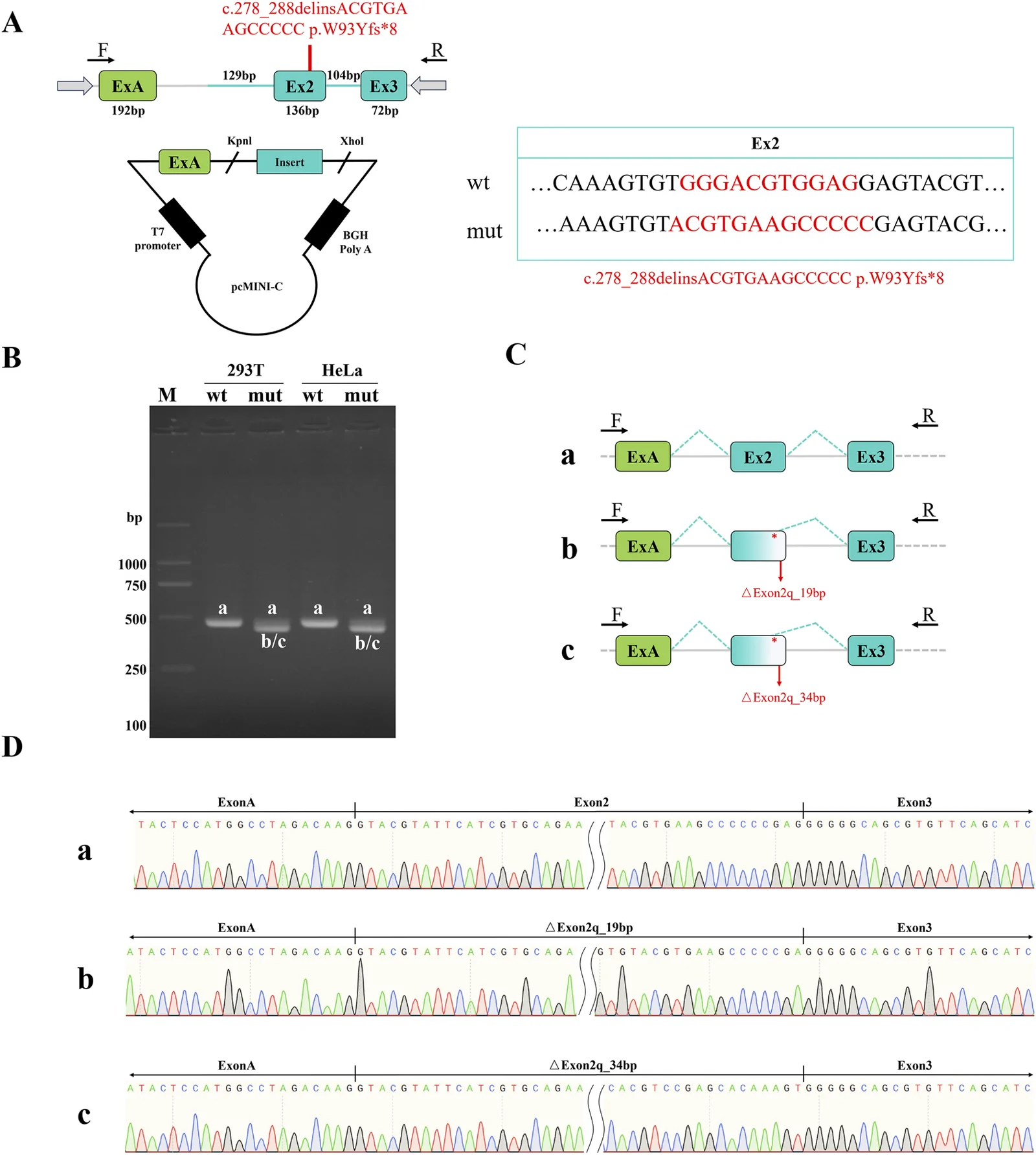
Minigene assay demonstrates variant-induced aberrant splicing of *P2RX2* variant. **(A)** Schematic illustration of the pcMINI-C minigene reporter construct. **(B)** Representative RT-PCR gel electrophoresis analysis in HEK293T and HeLa cells. The wt construct yields a single canonical transcript (band a). In contrast, the mutant construct generates two distinct aberrant splicing products (bands b and c, corresponding to the *mut1* and *mut2* transcripts, respectively) alongside the full-length transcript. **(C)** Schematic characterization of the splice isoforms. Band a corresponds to the canonical ExonA–Ex2–Ex3 transcript. Bands b and c represent isoforms with 3′truncations of exon 2 (deletion of 19 bp and 34 bp, respectively). **(D)** Sanger sequencing validation of the exon–exon junctions. Chromatograms confirm the specific deletions in bands b and c.

In silico predictions from three independent algorithms concurred with the minigene assay results. SpliceAI indicated that the complex indel disrupts canonical splice topology, yielding a wild-type donor loss score of 0.36 (at 32 bp) alongside a high cryptic donor gain score of 0.97 (at −2 bp; Table 1). Specifically, RDDCSC predicted an alternative splicing event resulting in a 36-bp deletion *via* a newly generated donor site, with the score increasing from 0.004 in the wild-type to 0.9623 in the mutant (p = 0.006). Similarly, BDGP identified cryptic donor sites near the mutation locus, yielding two donor gain scores of 0.49 (at 226–240 bp) and 0.68 (at 209–223 bp). To contextualize these findings, searching the SpliceVault database provided empirical support, revealing a naturally occurring cryptic donor site 19 bp upstream of the canonical site that is utilized at extremely low frequencies in healthy populations (detected in 116 GTEx and 217 SRA samples as a rare out-of-frame mis-splicing event). Together, our functional and predictive data indicate that the complex indel substantially upregulates this latent cryptic site, offering a mechanistic rationale for the 19-bp truncated transcript (*mut1*) observed *in vitro*.

**TABLE 1:** In silico splicing predictions and database validation for the *P2RX2* complex indel.

Tool	WT site score	Cryptic site score/Confidence	Evaluated splice effect
SpliceAI	Acceptor loss: 0.01 (at 145 bp) Donor loss: 0.36 (at 32 bp)	Acceptor gain: 0.06 (at −84 bp) Donor gain: 0.97 (at −2 bp)	Disruption of canonical donor site; activation of a new cryptic donor site
RDDCSC	Prediction score: 0.004	Prediction score 0.9623 (p = 0.006)	Deletion of 36 bp due to a new splicing donor site, resulting in a frameshift and premature termination
BDGP	Donor (at 243–257 bp)	Donor gain: 0.68 (at 209–223 bp) Donor gain: 0.49 (at 226–240 bp)	Activation of newly created cryptic donor sites near the mutation
SpliceVault	N/A	Documented in 116 GTEx and 217 SRA human samples	Utilization of a cryptic donor site located 19 bp upstream of the canonical site, resulting in a naturally occurring alternative splice product

### 3.4 *P2RX2* mRNA expression

To evaluate the stability of the aberrant transcripts, we quantified P2RX2 mRNA levels in HEK293T cells expressing the *wt*, *mut1*, or *mut2* constructs. RT-qPCR analysis revealed that both mutant isoforms exhibited significantly reduced mRNA abundance compared to the wild-type. Specifically, relative P2RX2 expression was decreased by approximately 48.3% for *mut1* (p < 0.05) and 69.4% for *mut2* (p < 0.01) compared to the wt (Figure 5A).

**FIGURE 5:**
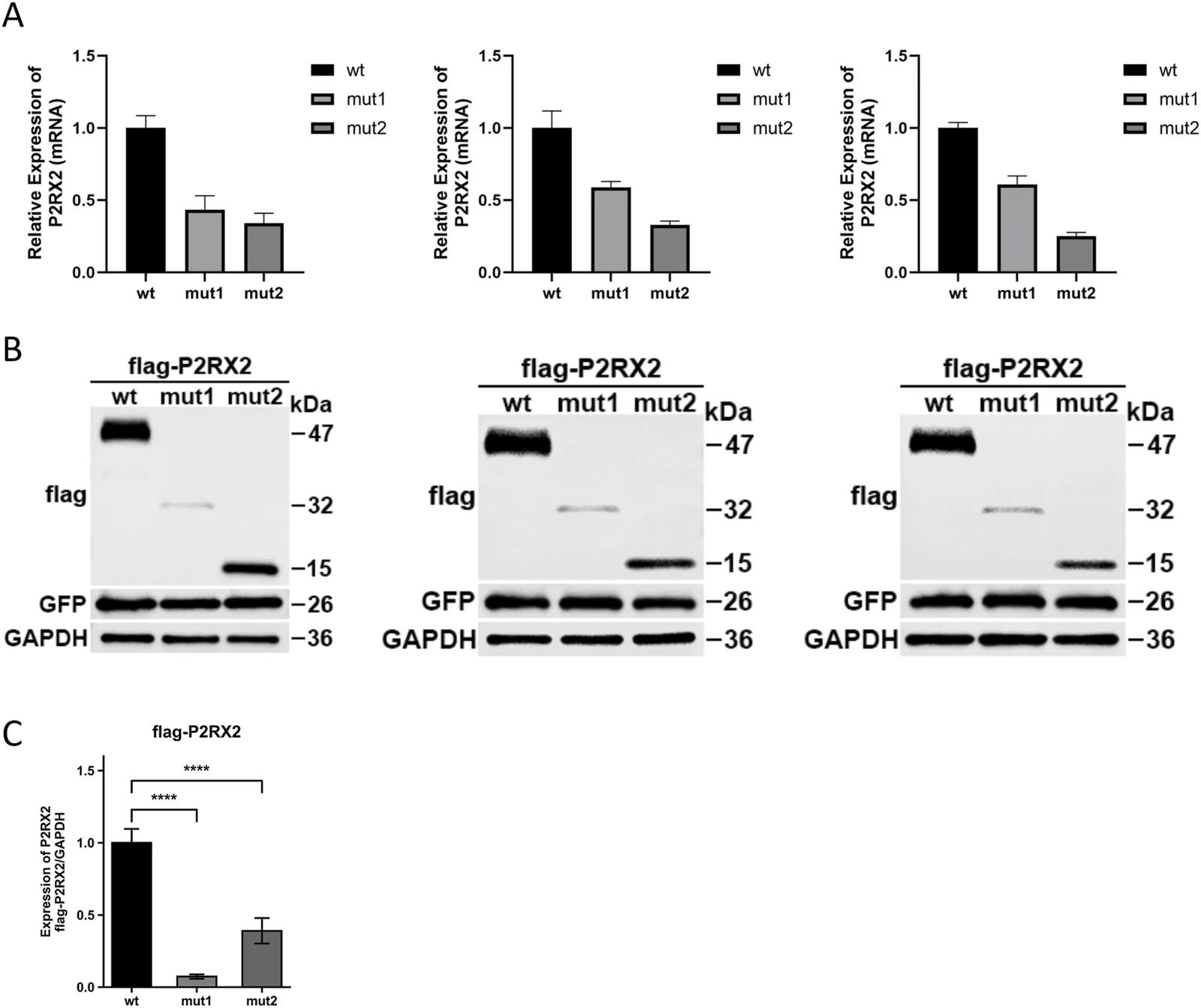
Reduced mRNA abundance and truncated protein expression of aberrant *P2RX2* splice variants. **(A)** RT–qPCR analysis of *P2RX2* mRNA levels in HEK293T cells transiently transfected with wt, *mut1*, or *mut2* constructs. **(B)** Representative Western blot of FLAG-tagged P2X2 isoforms. The wt construct yields the expected full-length protein at ∼47 kDa. In contrast, *mut1* and *mut2* generate truncated products migrating at ∼32 kDa and ∼15 kDa, respectively, confirming premature translation termination. Co-transfected GFP serves as a control for transfection efficiency, while GAPDH serves as a loading control. **(C)** Densitometric quantification of FLAG–P2X2 protein levels (normalized to GAPDH).

### 3.5 P2X2 protein expression

Immunoblot analysis revealed that the wt construct expressed a robust protein product migrating at ∼47 kDa, consistent with the expected size of full-length P2X2 (Figure 5B and supplemental figure). In contrast, cells transfected with the intronless cDNA constructs yielded distinct, truncated proteins corresponding to their predicted sizes and Flag-tag positions: *mut1* (c.278_309delinsACGTGAAGCCCCCGA; p.Trp93Tyrfs*173) migrated at ∼32 kDa, whereas *mut2* (c.276_309del; p.Trp93Glyfs*26) migrated at ∼15 kDa. Because the constructs were transfected independently, each lane displayed a single discrete band. However, despite the presence of detectable bands, quantitative densitometry (normalized to GAPDH) demonstrated that the steady-state protein levels of both mutants were markedly diminished compared to wt (Figure 5C). Specifically, protein abundance was reduced by 92.7% for *mut1* and 61.0% for *mut2* (p < 0.0001).

### 3.6 Transcriptomic profiling and network interaction analysis

Single-cell transcriptomic profiling of the murine cochlea unveiled spatially distinct yet complementary expression patterns for the target genes. *P2RX2* transcripts were predominantly mapped to sensory hair cells and spiral ganglion neurons, whereas *GJB2* showed robust enrichment within the supporting-cell syncytium, specifically in the inner sulcus and Deiters’ cells (Figure 6A). Despite this spatial segregation, functional enrichment analysis suggested convergence on shared biological themes, including ion transport, purinergic regulation, and cellular stress responses (Figure 6B). Notably, enrichment for calcium homeostasis terms motivated a hypothesis-generating network reconstruction to nominate potential connecting nodes. GeneMANIA analysis nominated *RYR3* (ryanodine receptor 3) as a candidate connector between the *P2RX2*-and *GJB2*-centered networks (Figure 6C). Given the established role of ryanodine receptors in calcium-induced calcium release and inner-ear calcium signaling, *RYR3* represents a biologically plausible node through which purinergic signaling and supporting-cell coupling could interface (Liang et al., 2009; Drescher et al., 2025); however, this inference is based on *in silico* functional associations and requires experimental validation.

**FIGURE 6:**
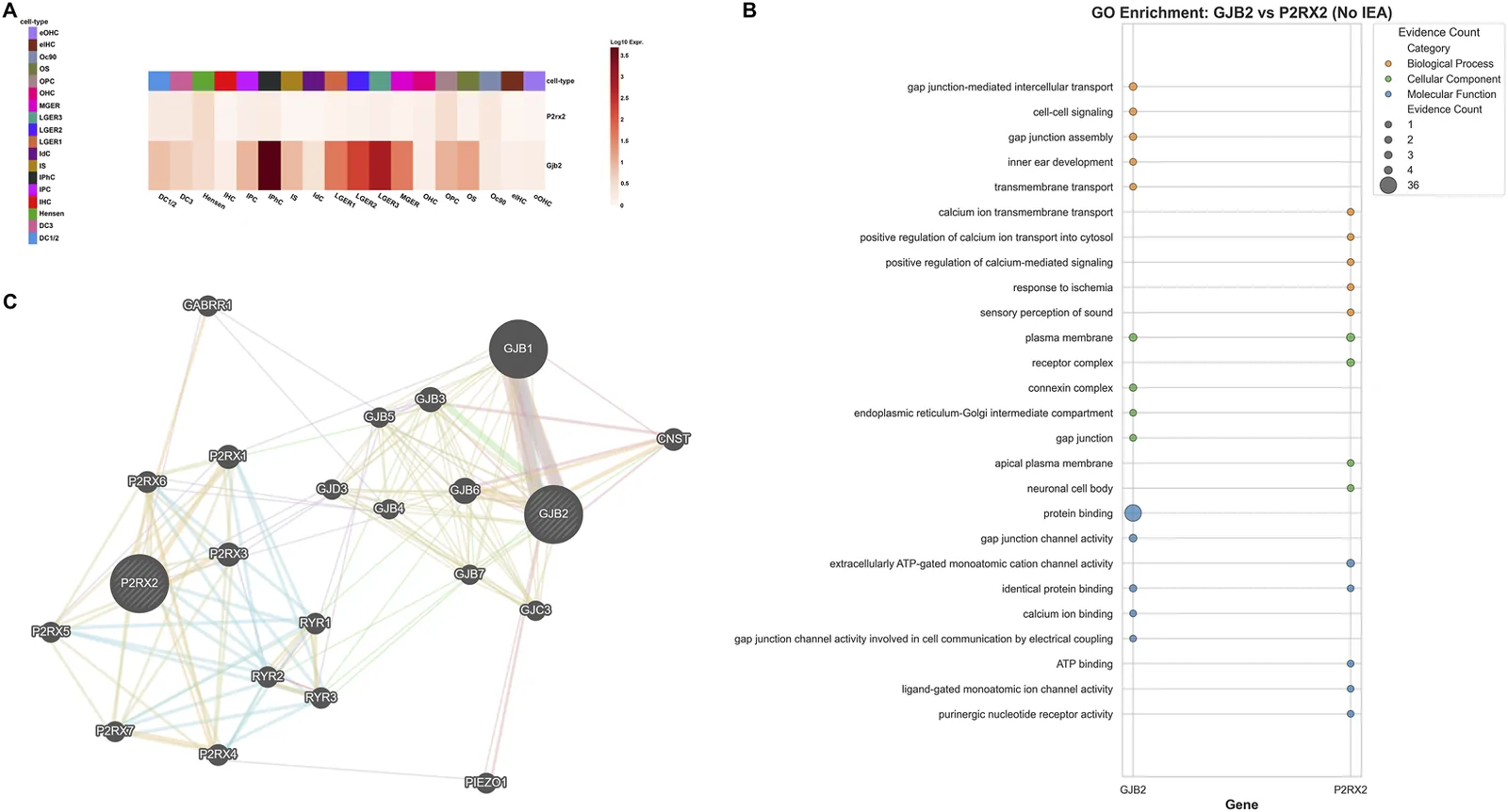
Integrated cochlear single-cell expression, functional enrichment, and network reconstruction of *P2RX2* and *GJB2*. **(A)** Transcriptomic landscape visualized *via* the gEAR portal. The heatmap displays log-transformed expression levels of murine orthologs *P2rx2* and *Gjb2* across annotated cochlear cell types. **(B)** Comparative Gene Ontology (GO) enrichment analysis for *GJB2* versus *P2RX2*. The bubble plot highlights complementary functional programs (filtered to exclude Inferred from Electronic Annotation [IEA] for high confidence). Dot size represents the evidence count, while colors denote the primary GO domain (Biological Process, Cellular Component, Molecular Function). **(C)** GeneMANIA interaction network centered on *P2RX2* and *GJB2*. The network highlights ryanodine receptors—most notably *RYR3*—as hypothesis-generating candidate nodes linking purinergic signaling and connexin-mediated intercellular coupling. Edges represent GeneMANIA-predicted functional associations, weighted by the underlying evidence.

## 4 Discussion

In this study, we report a complex indel in *P2RX2* and functionally define its molecular consequence. Minigene assays demonstrate cryptic splice usage generating two PTC-containing isoforms (*mut1*/*mut2*), and expression analyses show reduced steady-state mRNA and markedly diminished P2X2 protein output, establishing a *bona fide* LOF allele. Clinically, the pronounced intrafamilial variability—marked by severe early-onset hearing loss in the proband compared to a milder phenotype in the heterozygous father—is best explained by the intrinsic variable expressivity and incomplete penetrance characteristic of this *P2RX2* LOF variant. Although oligogenic or modifier effects remain conceptually possible, broader genetic interactions are currently speculative and require cautious interpretation.

The mutational landscape of *P2RX2* is characterized by a pronounced clustering in East Asian populations, comprising approximately 14 distinct variants across 78 individuals recorded to date (Yan et al., 2013; Faletra et al., 2014; Moteki et al., 2015; Chen et al., 2016; Cappi et al., 2020; Kaplanis et al., 2020; Wu et al., 2022; Ma et al., 2023; Antunes et al., 2024; Wang et al., 2024; Hao et al., 2025; Li et al., 2025). Most cases (73/78) were identified through hearing-loss cohorts; however, LoF alleles warrant separate consideration because genotype classification and phenotype attribution are not always aligned. Among seven published LoF variants, several truncating or splice-disrupting alleles—c.121dup (p.Leu41ProfsTer231), c.864dup (p.Asp289Ter), and c.906-1G>A—have been reported in affected individuals or families (Boucher et al., 2020; Antunes et al., 2024; Wang et al., 2024), supporting a potential pathogenic contribution. Notably, the exon two complex indel observed in our family (c.278_288delinsACGTGAAGCCCCC; commonly annotated as p.Trp93TyrfsTer8) was also reported in a newborn genetic screening study in which the infant had a family history of hearing loss (Hao et al., 2025). This clinical vignette closely parallels our proband, who passed initial newborn hearing screening but subsequently experienced delayed-onset, profound sensorineural hearing loss. This trajectory suggests that individuals harboring *P2RX2* LoF variants may possess sufficient cochlear functional reserve at birth, only to manifest progressive auditory decline over time. Nevertheless, in the absence of complete pedigree data, segregation analysis, and longitudinal audiologic tracking, the true clinical penetrance of such variants remains challenging to establish. Consequently, these diagnostic and evidence gaps obscure clinical interpretation: while LoF alleles readily meet criteria for high-level pathogenic classification under the ACMG/AMP framework (e.g., PVS1, PM2), their precise contribution to hearing impairment is often confounded by variable expressivity, incomplete penetrance, or undetected co-occurring variants in secondary deafness genes.

Recent high-resolution cryo-EM studies reveal that P2X2 undergoes extensive conformational reorganization upon ATP binding, transitioning from a closed apo “teepee-shaped” architecture to an open, desensitized state (Westermann et al., 2025). This activation relies on precise salt-bridge switching (e.g., Arg290–Glu167) and the mobility of the “left flipper” domain to gate ATP access. Physiologically, these structural dynamics underpin the receptor’s role as a “protective shunt,” enabling the rapid damping of cochlear potential to prevent excitotoxicity during acoustic stress. Structurally, the aberrant isoforms of our study are predicted to terminate prematurely, truncating the receptor upstream of the essential transmembrane topology (TM1 and TM2). This truncation abolishes the molecular machinery required for ATP binding, ion pore formation, and channel gating. Lacking these integral structural domains, the nascent peptides are likely flagged as misfolded substrates and targeted for rapid clearance *via* Endoplasmic Reticulum-Associated Degradation. This mechanism aligns with our Western blot data, which showed markedly diminished protein output rather than the accumulation of truncated fragments.

The striking phenotypic discordance observed across animal models offers a compelling framework for interpreting the variable severity of *P2RX2* deficiency. While *P2rx2*-knockout mice exhibit preserved baseline thresholds, with vulnerability unmasked only by acoustic trauma or aging (Yan et al., 2013), a recent *p2rx2*-null zebrafish model reveals a far more severe pathology. In zebrafish, P2X2 deficiency drives overt baseline hearing loss and spontaneous hair cell reduction even in the absence of external stress (Zhang et al., 2025). Notably, objective audiologic testing in our proband during early childhood demonstrated profound bilateral dysfunction (absent OAEs and absent ABR responses at high stimulus levels), with later behavioral audiometry confirming severe-to-profound thresholds across all frequencies. Two clinical aspects of this phenotype merit explicit discussion. First, the involvement of low frequencies is not incompatible with DFNA41 biology *per se*, as low-frequency impairment has been documented in noise-exposed DFNA41 carriers and experimental noise paradigms in P2rx2-deficient mice (Yan et al., 2013; Chen et al., 2021). Second, the directionally severe phenotype in our proband—consistent with the zebrafish model—contrasts with classic DFNA41 missense pedigrees that typically present in the second decade. This discrepancy suggests that the clinical impact of reduced P2X2 output may be influenced by: (i) a stronger molecular impact of a true LoF allele compared to missense alleles; (ii) unmeasured environmental or perinatal contributors (e.g., ototoxic exposures, subclinical infections); and/or (iii) co-occurring genetic modifiers in secondary deafness loci (Moteki et al., 2015; Boucher et al., 2020).

In this context, the co-inheritance of a heterozygous *GJB2* c.235delC allele in the proband warrants careful evaluation. Because *GJB2* c.235delC is an autosomal recessive allele and heterozygous carriers (including the mother and sister in this pedigree) are typically asymptomatic, attributing the proband’s severe phenotype solely to a *GJB2* modifier effect lacks direct statistical support. However, from a physiological perspective, *GJB2* (Cx26) and *P2RX2* occupy distinct but functionally convergent positions within the cochlear homeostatic network, specifically bridging purinergic signaling and potassium (K^+^) recycling (Figure 7). Cx26 forms hemichannels that serve as conduits for stimulus-evoked ATP release (governing extracellular ATP supply) alongside gap junctions essential for intercellular K^+^ transport. Concurrently, P2X2 receptors in hair cells and spiral ganglion neurons act as downstream ATP sensors that facilitate ATP-gated “K^+^ sinking” and protective responses (Anselmi et al., 2008; Yan et al., 2013; Chen et al., 2022; Wand et al., 2025). In this integrated supply-versus-sensing framework, a null GJB2 allele could subtly attenuate supporting-cell ATP release and gap-junctional coupling. When coupled with P2X2 receptor haploinsufficiency, which blunts ATP-sensing and downstream intracellular calcium (Ca^2+^) dynamics (e.g., *via* nodes like *RYR3*), this dual impairment theoretically leads to toxic K^+^ accumulation and diminishes cochlear homeostatic reserve. Nevertheless, because single-cell transcriptomics confirm that P2RX2 and GJB2 are spatially segregated across different cell types, and because no prior direct digenic association has been established in human cohorts, this purinergic supply-sensing model must be framed strictly as a speculative working hypothesis. The observed intrafamilial variability remains most parsimoniously explained by the intrinsic variable expressivity and incomplete penetrance of the *P2RX2* LoF allele itself.

**FIGURE 7:**
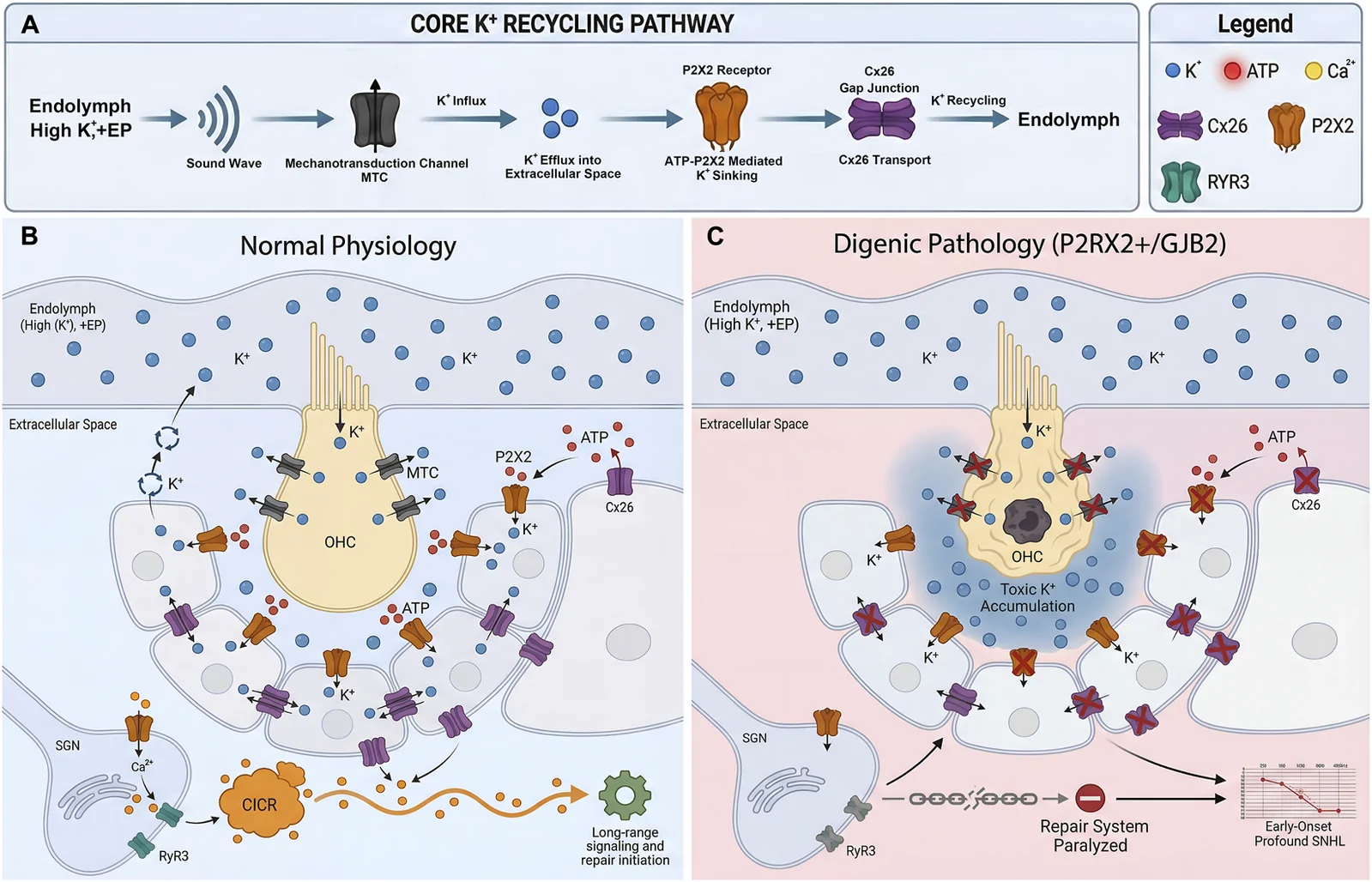
Hypothetical Conceptual Model of Potential *P2RX2* and *GJB2* Functional Convergence in Cochlear Homeostasis. **(A)** The cochlear potassium recycling pathway. **(B)** Physiological purinergic–calcium signaling. Under normal conditions, sound stimulation triggers ATP release into the endolymph, partially mediated by Connexin 26 (Cx26) hemichannels. Extracellular ATP activates P2X2 receptors on Hair Cells and Spiral Ganglion Neurons, driving an initial Ca^2+^ influx. This influx recruits Ryanodine Receptor 3 (RyR3) on the Endoplasmic Reticulum. **(C)** Hypothetical Dual-Pathway Vulnerability (*P2RX2* +/−; *GJB2* +/−). In the dual-variant carrier, the homeostatic circuit is dismantled by a “double hit.” First, *GJB2* haploinsufficiency diminishes the ATP trigger. Second, *P2RX2* haploinsufficiency limits the receptor’s capacity to respond. This combined deficit results in a sub-threshold Ca^2+^ signal that fails to activate the RyR3-mediated amplification loop (CICR failure). Consequently, K^+^ clearance is compromised, leading to toxic extracellular K^+^ accumulation, hair cell cytotoxicity, and the observed early-onset, profound sensorineural hearing loss.

Several limitations merit consideration. First, the clinical analysis is constrained by a single pedigree with one double-variant proband (n = 1), which is methodologically insufficient for causal inference regarding digenic inheritance. Furthermore, complete quantitative audiometric data for the father were unavailable because he is a military service member stationed away from home, restricting our phenotypic comparison to qualitative clinical history and preventing direct testing of the “noise-dependent penetrance” hypothesis in this pedigree. Second, unmeasured environmental risk factors (such as differential noise exposure or ototoxic history) and unscreened genetic modifiers cannot be ruled out. Third, our functional assays—based on a minigene system and heterologous expression—confirm the splicing defect and reduced P2X2 output but do not fully recapitulate the complex native cochlear microenvironment. Finally, the theoretical interplay between *GJB2* and *P2RX2* is inferred from physiological models and *in silico* network analyses; this link remains purely hypothesis-generating and requires rigorous validation in larger, multi-center cohorts and dedicated *in vivo* co-expression models.

In summary, we identified and functionally characterized a complex indel in *P2RX2* exon two in a Han Chinese family. Minigene and expression assays demonstrate that the variant disrupts splicing, producing unstable transcripts and markedly reduced P2X2 output, consistent with haploinsufficiency. The pronounced intrafamilial variability is most parsimoniously attributed to the variable expressivity and incomplete penetrance inherent to *P2RX2* loss-of-function alleles, while potential modifying roles of secondary variants remain speculative. These findings broaden the DFNA41 mutational spectrum and emphasize the importance of considering non-monogenic expressivity modifiers and detailed environmental histories when evaluating hereditary hearing loss.

## Data Availability

The datasets presented in this study can be found in online repositories. The names of the repository/repositories and accession number(s) can be found in the article/Supplementary Material.
