# Supplementary material for: A P2RX2 exon-2 complex indel disrupts splicing and reduces P2X2 output: implications for DFNA41 and variable expressivity
Source: Front Genet. 2026 Oct 20;17:1934538. doi: 10.3389/fgene.2026.1934538 (PMC13727070; doi:10.3389/fgene.2026.1934538)

Raw Western blots for flag-P2RX2, GFP, and GAPDH (corresponding to main text Figure 5B). Full-length, unprocessed membranes are shown with all physical edges clearly visible, demonstrating the unprocessed nature of the blots. The target proteins (flag-P2RX2: wt, mut1, mut2)) and the loading control (GFP, GAPDH) are labeled accordingly. Molecular weight ladder markings (kDa) are annotated on the left of each blot.

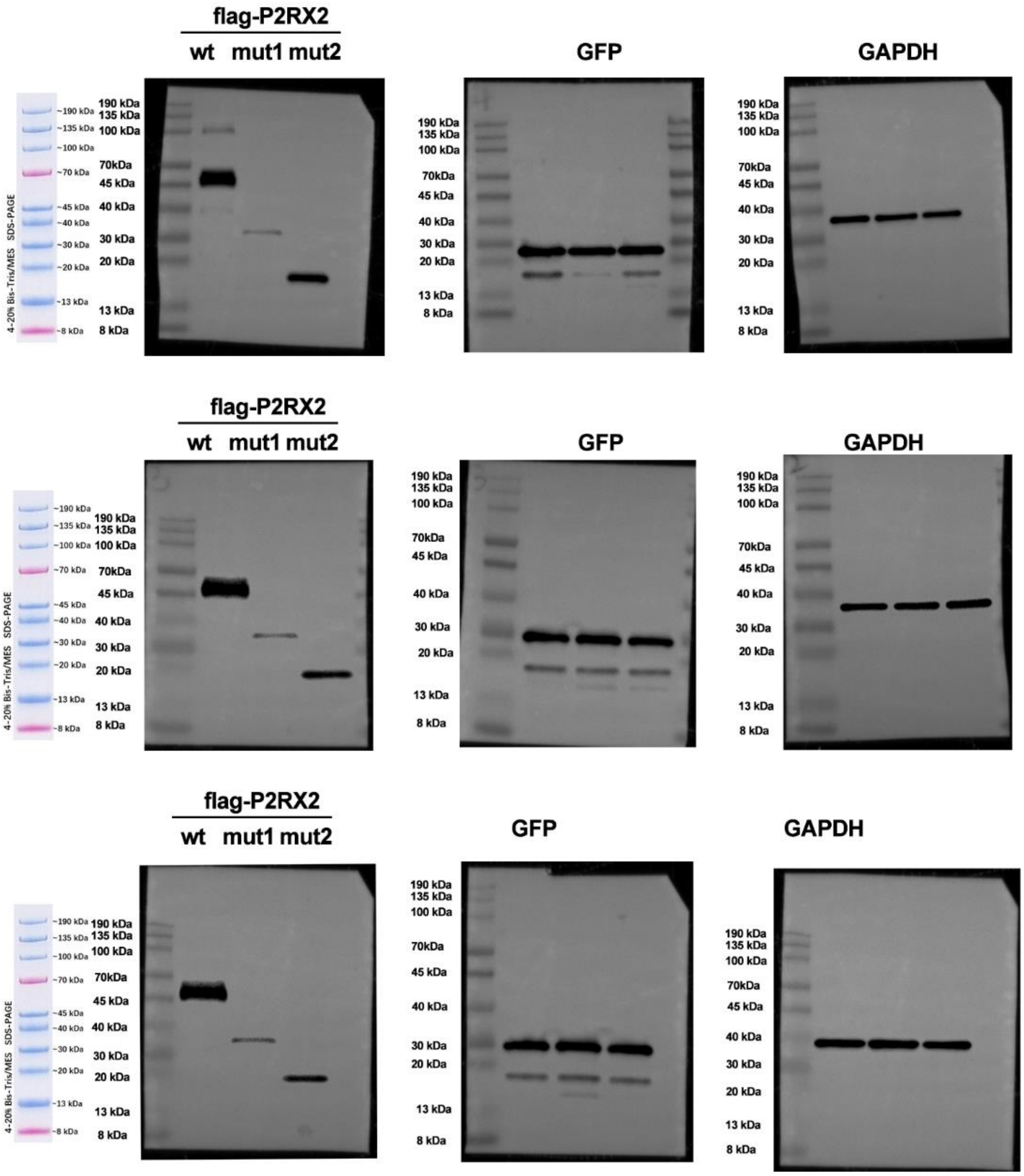

Raw agarose gel electrophoresis (corresponding to main text Figure 4B). The full-length, unprocessed gel image displays the amplification products for the wt and mut groups in 293T and HeLa cell lines. DNA ladder markings (bp) are clearly indicated in the marker lane (M).

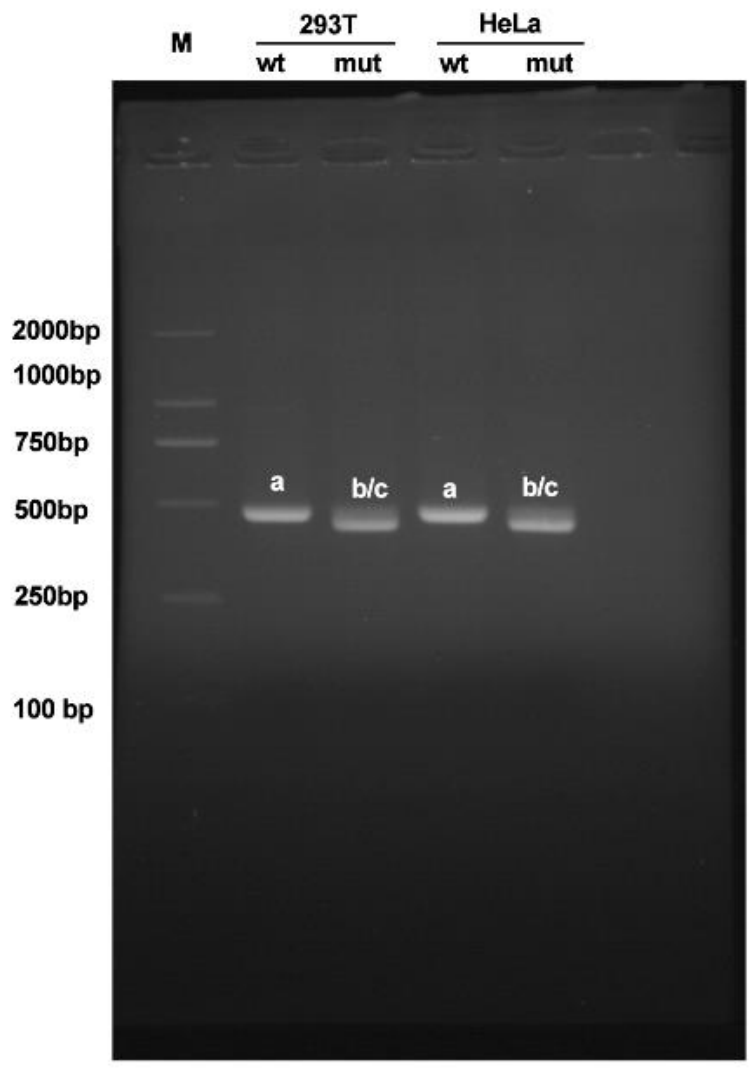

Supplement: Supplementary file 1 [file DataSheet1.pdf]
