# Supplementary material for: A P2RX2 exon-2 complex indel disrupts splicing and reduces P2X2 output: implications for DFNA41 and variable expressivity
Source: Front Genet. 2026 Oct 20;17:1934538. doi: 10.3389/fgene.2026.1934538 (PMC13727070; doi:10.3389/fgene.2026.1934538)
Supplement: Supplementary file 2 [file Table1.docx]

**Supplementary Table S1. Sequences of primers and details of antibodies used in this study.**

|  | primers | Primer sequences (5′-3′) |
| --- | --- | --- |
| Mini-gene | pcMINI-C-P2RX2-KpnI-F | ggtaGGTACCagggcgcggggcaggcccca |
|  | pcMINI-C-P3RX2-XhoI-R | TAGACTCGAGCTCGGGGCAGGTTCCCTGGG |
|  | pcMINI-P2RX2-KpnI-F | ggtaGGTACCagggcgcggggcaggcccca |
|  | pcMINI-P2RX2-XhoI-R | tttcCTCGAGgctcagaaagagggggcggt |
|  | P2RX2-mut-F | CCGAGCACAAAGTGTACGTGAAGCCCCCGAGTACGTGAAGCCC |
|  | P2RX2-mut-R | GGGCTTCACGTACTCGGGGGCTTCACGTACACTTTGTGCTCGG |
|  | pcMINI-F | ACTTAAGCTTatgagtgggctttggggtggccggtt |
|  | pcMINI-R | TAGAAGGCACAGTCGAGG |
|  | pcMINI-C-F | ACTTAAGCTTatgagtgggctttggggtggccggtt |
|  | pcMINI-C-R | TAGAAGGCACAGTCGAGG |
| Vector construction | pCMV-3xFlag-Neo(EGFP)-P2RX2-HindIII-F | tgacaagcttATGGCCGCCGCCCAGCCCAA |
|  | pCMV-3xFlag-Neo(EGFP)-P2RX2-EcoRI-R | cgatgaattcTCAGAGTTGAGCCAAACCTT |
|  | P2RX2-mut1-F | CCGAGCACAAAGTGTACGTGAAGCCCCCGAGGGGGCAGCGTGTTC |
|  | P2RX2-mut1-R | GAACACGCTGCCCCCTCGGGGGCTTCACGTACACTTTGTGCTCGG |
|  | P2RX2-mut2-F | GGATCACCACGTCCGAGCACAAAGTGGGGGCAGCGTGTTCAGCATCATCA |
|  | P2RX2-mut2-R | TGATGATGCTGAACACGCTGCCCCCACTTTGTGCTCGGACGTGGTGATCC |
| Q-PCR | P2RX2-qPCR-F | AACCCCAAGTACTCCTTCCG |
|  | P2RX2-qPCR-R | ATGGTGGGAATCAGGCTGAA |
|  | H ACTIN-F | TGACGTGGACATCCGCAAAG |
|  | H ACTIN-R | CTGGAAGGTGGACAGCGAGG |
